# An observational study of spectators’ step counts and reasons for attending a professional golf tournament in Scotland

**DOI:** 10.1136/bmjsem-2017-000244

**Published:** 2017-07-21

**Authors:** Andrew D Murray, Kieran Turner, Daryll Archibald, Chloe Schiphorst, Steffan Arthur Griffin, Hilary Scott, Roger Hawkes, Paul Kelly, Liz Grant, Nanette Mutrie

**Affiliations:** 1 Physical Activity for Health Research Centre, University of Edinburgh, Edinburgh, UK; 2 Department of Sport and Exercise, University of Edinburgh, Edinburgh, UK; 3 Scottish Collaboration for Public Health Research and Policy, University of Edinburgh, Edinburgh, UK; 4 European Tour Golf, Virginia Water, UK; 5 College of Medical and Dental Sciences, University of Birmingham, Birmingham, UK; 6 Robert Gordon University, Aberdeen, UK; 7 Sports and Exercise Medicine, University College London, Birmingham, UK; 8 Global Health Academy, University of Edinburgh, Edinburgh, UK

**Keywords:** physical activity, golf, health, spectating, public health

## Abstract

**Background:**

Spectators at several hundred golf tournaments on six continents worldwide may gain health-enhancing physical activity (HEPA) during their time at the event. This study aims to investigate spectators’ reasons for attending and assess spectator physical activity (PA) (measured by step count).

**Methods:**

Spectators at the Paul Lawrie Matchplay event in Scotland (August 2016) were invited to take part in this study. They were asked to complete a brief questionnaire with items to assess (1) demographics, (2) reasons for attendance and (3) baseline PA. In addition, participants were requested to wear a pedometer from time of entry to the venue until exit.

**Results:**

A total of 339 spectators were recruited to the study and out of which 329 (97.2%) returned step-count data. Spectators took a mean of 11 589 steps (SD 4531). ‘Fresh air’ (rated median 9 out of 10) then ‘watching star players’, ‘exercise/physical activity’, ‘time with friends and family’ and ‘atmosphere’ (all median 8 out of 10) were rated the most important reasons for attending.

**Conclusion:**

This study is the first to assess spectator physical activity while watching golf (measured by step count). Obtaining exercise/PA is rated as an important reason for attending a tournament by many golf spectators. Spectating at a golf tournament can provide HEPA. 82.9% of spectators achieved the recommended daily step count while spectating. Further research directly assessing whether spectating may constitute a ‘teachable moment’, for increasing physical activity beyond the tournament itself, is merited.

## Background

Researchers, policy-makers and practitioners concur that regular physical activity (PA) benefits persons of all ages and backgrounds. It has positive effects on mental health, physical health and longevity for both individuals and populations.^1–4^

A recent aim of major sporting events has been to secure a legacy of increased PA or participation in sport following the event.^5^ Major multi-sport games have failed to achieve an inherent, substantial PA legacy.^6^ Measures that could help address this lack of legacy include (1) producing a clear strategy to increase participation and (2) de-emphasising the sporting element and promoting PA more generally (for example, walking) rather than simply the sport being played.^7 8^

Golf can provide a novel and suitable narrative to provide a link between sport, walking and potential health benefits.^9^ Golf playing and spectating is particularly popular in middle-aged and older adults in North America, Europe and Asia in particular.^10^ This demographic typically have lower levels of PA compared with younger adults and children.^11–13^

Collectively, tournaments in the USA alone can draw over 10 million spectators per year.^14^ Those watching the action at several hundred tournaments on six continents worldwide may have the opportunity to gain health-enhancing physical activity (HEPA) on the available square miles of playing arena.^11^ Indeed, the existing literature suggests that golf spectators rate perceived ‘health benefits’ and ‘exercise’ as important considerations in attending tournaments,^15–17^ with Lyu and Lee segmenting the motivations of spectators into ‘excitement seekers’, ‘exercise seekers’, ‘interest seekers’ and ‘escape seekers’.^17^ Our recent scoping review identified knowledge gaps, namely that no studies have characterised the effects of spectating at golf tournaments on PA knowledge or PA levels.^9 18^ We aim to contribute to these knowledge gaps. We first address critical feasibility questions and assess the extent to which spectating delivers opportunities for PA.

Our research questions were the following:

1. Is studying spectator PA through pedometer measured step counts feasible at a professional golf tournament?
2. What reasons do spectators at a European Tour event identify for their attendance?
3. Can spectators gain a relevant dose of PA (measured by step count) while attending a professional golf tournament?

## Methods

We conducted a cross-sectional study consisting of two linked elements: a questionnaire completed by spectators on entering the course and a measure of step count from the time a spectator entered the venue until the time they exited. Ethical approval was granted (15 July 2016) by the Moray House School of Education Ethics Committee at the University of Edinburgh.

### Data collection

Data were collected on all days of tournament play (4–7 August 2016) at the European Tour Paul Lawrie Matchplay event in Scotland. The European Tour meteorological service recorded temperatures of between 18°C and 21°C (highs) and 9°C–13°C (lows). Winds were light to moderate, except on the final day of play where 40–45 miles/hour gusts were experienced. Rain fell for <10% of the duration of play.

Spectators attending the event were approached by one of six trained researchers who invited spectators to read a two-page participant information sheet, detailing the purposes of the study as they arrived. Those willing to partake were assessed against the inclusion and exclusion criteria stated in table 1 below by a researcher.

**Table 1:** Inclusion and exclusion criteria

Inclusion criteria	Exclusion criteria
Spectators at the European Tour Paul Lawrie Matchplay Aged ≥18 years Able to walk (walking aids permitted) Unstable cardiovascular disease not reported	Non-spectators (for example staff, marshals, players, caddies) Spectators that had taken part in the study on previous days Aged under 18 years Inability to walk Reported unstable cardiovascular disease (critical aortic stenosis, unstable angina, myocardial infarction within 6 weeks—a medical doctor was part of the research team and could provide individual case advice)

Those eligible were invited to sign a consent form, and following this completed a baseline questionnaire. This questionnaire was devised from a review of relevant previous studies^15–17 19^ and was refined following discussion with the research team and officials from the European Tour golf. The full questionnaire is shown in the online supplementary appendix 1 and included seven demographic items, eight items including a free text option assessing reasons for spectating, and three items assessing self-reported current PA levels and interest in becoming more physically active. These last three items were facilitated by a member of the research team using a validated tool (Scot-PASQ; NHS Health Scotland, UK).

10.1136/bmjsem-2017-000244.supp1 Supplementary Appendix 1

Following this, a researcher fitted a Silva Ex Step (Silva, Stockholm, Sweden) pedometer to the lateral aspect of the right hip region of each participant, noting the time this was fitted. Participants were asked to check the pedometer was registering steps after 1–2 min, and if not it was repositioned to an adjacent position. The European Tour works with a Scottish charity that champions walking ‘Paths for All’. Paths for All recommended the Silva Ex Step as having high usability compared with other devices. A brief validation of five Silva Ex Step devices was performed with <5% difference for all devices noted compared with Actigraph (Pensacola, Florida, USA). Paths for All also offered all spectators information relating to spectating and health, as is standard at Scottish-based European Tour events.

The participant then spectated for a length of time of their choosing and in a manner of their choosing. Prior to exiting the venue, participants returned the pedometer to a member of the research team who checked and recorded the number of steps taken and the time returned.

### Data analysis

With regard to feasibility, we decided, rather than to specify in advance a hypothesis to determine feasibility, that we would assess feasibility on a subjective basis based on response, recruitment, compliance and the human and equipment resources required.

Pedometer failure is a recognised issue in step-count studies. We had specified criteria for inclusion and exclusion of data. When pedometers were returned, the values were entered into the database, and the researcher assessed them for face validity. The participant sometimes offered information unprompted that the pedometer had failed. Where there was clear error, the result was excluded.

Statistical Package for the Social Science V.22 software was used for data management and analysis. Variables were assessed for normality with means or medians reported as appropriate. We used independent samples t-tests to explore any possible differences in step counts by age and gender. The association between minutes spectating and steps taken was tested using Pearson correlation coefficient.

## Results

### Feasibility/spectator characteristics

European Tour figures show the 2016 Paul Lawrie Matchplay was attended by 1500 paying spectators. Approximately 600 spectators in total were approached to take part in the study. A total of 339 spectators were recruited to the study and agreed to complete the questionnaire. Of those who did not agree to take part, most indicated that they were in a hurry to go and watch the golf. Of these 339 participants, 329 collected step count data and returned the pedometer (97.2%). Twenty (6.1%) pedometers failed to register accurate readings. Participants recruited and completing the study represented 22.6% of the eligible tournament population. While not part of the study, researchers were approached by marshals, children, golf caddies, professional players and returning spectators requesting literature relating to golf and health and/or pedometers to monitor their step count highlighting interest in this topic beyond direct participants.

The baseline characteristics of participants are shown in table 2. Approximately two-thirds of participants were men, with men between 40 and 59 years old most strongly represented.

**Table 2:** Baseline characteristics of participants

Age (years)	Men	Women	Total
18–39	49	18	67
40–59	105	46	151
≥60	68	43	111
Total	222	107	329

### Reasons for attendance

Within the baseline questionnaire, participants were asked to rate reasons for spectating on a scale of 1 (of no importance) to 10 (of extremely high importance).

Median and mode values showing spectators’ stated reasons for attendance are shown in Table 3. ‘Fresh air’ (rated median 9 out of 10) then ‘watching star players’, ‘exercise/physical activity’, ‘time with friends and family’ and ‘atmosphere’ (all median 8 out of 10) were rated the most important reasons for attending (table 3).

**Table 3:** Reasons for attendance at the Paul Lawrie Matchplay as rated by participants on entry to the venue

	Watch star players	Learn from star players	Non-golfing entertainment	Atmosphere	Fresh air	Exercise/physical activity	Time with friends/family
No of respondents	338	337	333	332	333	334	334
Median	8.00	7.00	5.00	8.00	9.00	8.00	8.00
IQR	2	4	5	2	3	4	3
Mode	8	8	1	8	10	10	10

**Figure 1:**
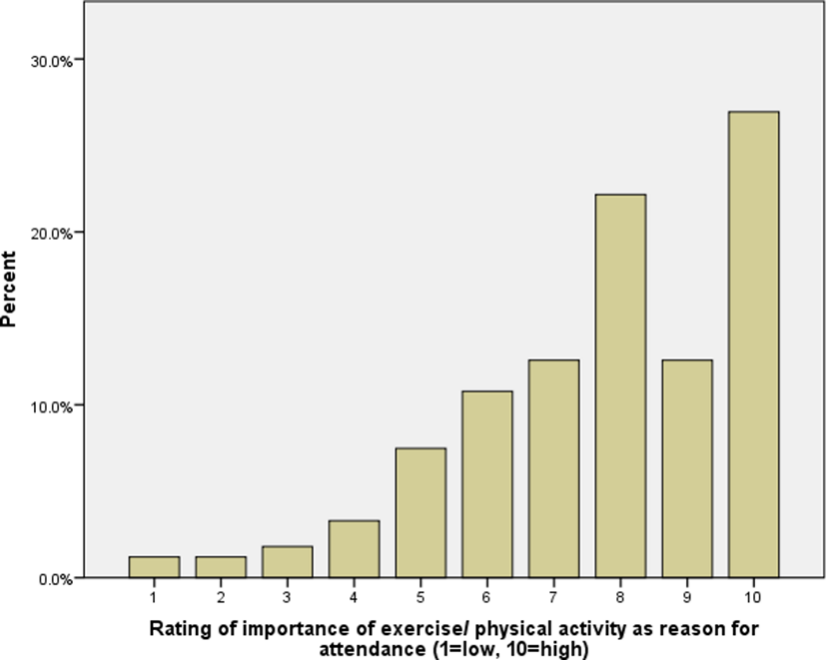
Participant rating (1–10) of ‘exercise/physical activity’ as a reason for attendance on entry to the venue.

In terms of the importance of reasons for attendance, exercise and physical activity was of interest to this paper on spectator health. The relative percentage for spectator rating of importance of exercise/physical activity as a reason for attending is displayed in figure 1.

### Measured spectator PA

Table 4 shows the mean/median number of steps taken by spectators, stratified by gender. The independent samples t-test revealed a statistically significant difference by gender with men taking approximately 1858 more steps on the day they attended (95% CI 784 to 2933, p<0.001). There were no important differences in step counts by age group.

**Table 4:** Mean/median number of steps taken by gender

Gender	Measure		Statistic
Men	Mean		12 172.5
	95% CI for mean	Lower bound	11 586.6
		Upper bound	12 758.4
	Median		11 362.5
	SD		4327.6
	Minimum		1576
	Maximum		25 312
Women	Mean		10 314.1
	95% CI for mean	Lower bound	9361.9
		Upper bound	11 266.2
	Median		10 039.0
	SD		4724.2
	Minimum		310
	Maximum		25 098

**Figure 2:**
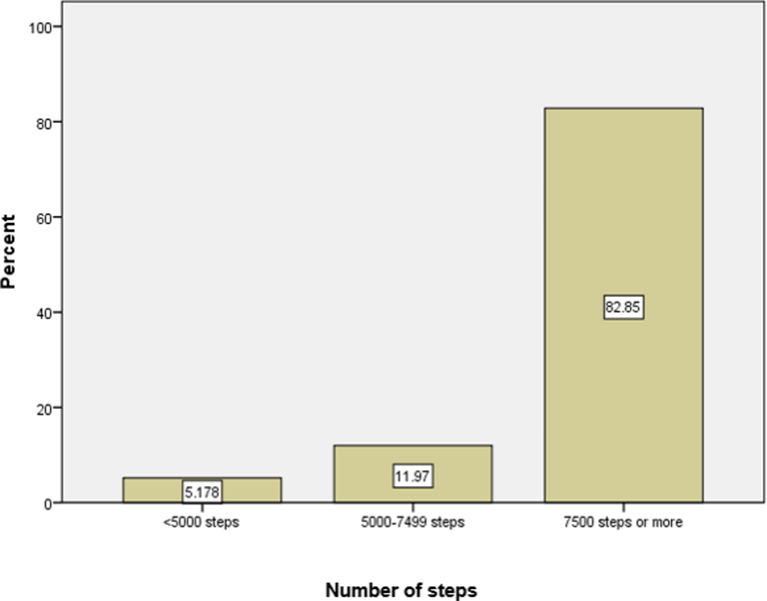
Number of spectators: <5000 steps, 5000–7500 steps and >7500 steps.

Figure 2 displays the number of steps taken categorised into (1) inactive, (2) low active and (3) meeting moderate to vigorous physical activity guidelines.^20^

### Number of steps

An increasing number of minutes spectating had a moderate association with total steps taken (r=0.67). This shows that on average, participants attending for longer accrued more steps.

Questionnaire data from the SCOT PAS-Q items collected as participants entered the venue highlighted that 89.3% reported meeting the aerobic moderate to vigorous physical activity guidelines in the previous week, while 68.1% reported being ‘interested in being more physically active’.

## Discussion

### Principal findings

#### Feasibility

This study indicates that it is feasible to study adult spectator PA (by pedometer-measured step counts) at a professional golf tournament. Approximately 56% of spectators approached agreed to participate, and of these, 97.2% returned questionnaire and step-count data.

It should be noted that it was not practical to engage with every spectator who entered the venue. Spectators can typically access the venue through more than one entrance and often arrive in groups having largely travelled by coach transfer. A larger number of researchers would be needed to engage with a larger volume and proportion of attending spectators.

### Spectator reasons for attending

Spectators rated a number of reasons for attending this professional golf tournament as highly important. ‘Watch star players’, ‘atmosphere’, ‘fresh air’, ‘exercise/physical activity’ and ‘time with friends and family’ all scored median and mode values of 8 out of 10 or greater. Importantly, for this work, obtaining exercise/PA can be a motivation for attending for participants at this event. The median rating was 8 out of 10, with a mode of 10, representing ‘of extremely high importance’.

### The extent to which spectating delivers an opportunity for PA

This is the first published study to measure golf spectator PA by step count. Data show participants took a mean of 11 589 (SD 4531, range 25 002) and a median of 11 086 steps. Through spectating alone, 82.9% of participants met Tudor-Locke *et al*’s daily guidelines indicative of a ‘physically active lifestyle’ from activity while spectating with 94.8% of spectators meeting either ‘low active’ or ‘physically active’ lifestyle.^20^ As may be expected, an increasing number of minutes spectating had a positive association with increased total steps taken. There were no apparent differences in step counts by age group, but there was a statistically significant and potentially clinically relevant difference by gender with male participants taking approximately 1858 more steps per day than female participants.

### Comparison to the literature and explanation for findings

#### Spectator reasons for attending

A large body of research has assessed spectator motivations for attendance at sporting events, but most of these pertain to team-based sports,^14^ with data specific to golf limited.^14–17^ McDonald *et al* found clear spectator motivation differences between golf spectators and spectators of other sports.^21^

Watching star players is the most powerful motivator for golf spectator attendance in most previous studies conducted,^14–17^ and the current study supports the importance spectators place on this. Robinson *et al* argue that the prime marketing focus for events should be on specific well-recognised golfers playing.^14^ However, spectators in our sample rate at least equally highly other reasons for spectating including ‘fresh air’, ‘spending time with friends and family’ and ‘exercise/physical activity’. These data support Lyu and Lee’s assertion that factors such as these offer attractive marketing angles to tournament organisers/promoters, with the aim of increasing spectator volume and engagement.^17^

These factors were not probed as explicitly in Robinson *et al*’s study of US spectators, with the questionnaire employed not golf specific. It is known motivations for golf spectators are different to team sports, being broader and less homogeneous.^21^

### Spectator attitudes towards changing exercise/PA

Evidence from North America, Asia and Europe is consistent and growing that exercise/PA can be a motivator for attending golf tournaments.^15–17^ Golf tournaments and their spectators are heterogeneous, and some may be more motivated than others by PA benefits based on individual, cultural, climactic and tournament differences. They may also be likely to be meeting minimum PA levels already. Our study did not find significant age-related and gender-related differences in attitudes of spectators towards exercise/PA. The literature broadly supports a greater emphasis of these benefits by event promoters,^15–17^ which may be beneficial in terms of engagement with spectators, local communities and funding organisations.

### PA gained while spectating

There are no previous published studies that measured the levels of PA attained by golf spectators. Unpublished data (obtained via personal correspondence, Event Scotland) from the 2014 Ryder Cup, Gleneagles, UK, show over 20 000 spectators tagged every checkpoint at locations on course, indicating they had walked 8 kilometres each, or 100 000 miles collectively. At the 2016 Shenzhen Open, Shenzhen, China, 6500 spectators completed a ‘health walk’ intervention, of 10 km each, adding up to a distance seven times the length of the Great Wall of China (personal communication, Shenzhen Open).

Step counting using pedometers is a well-established method of measuring PA by the general public, researchers and policy-makers.^20^ Data showed that 82.9% of participants met Tudor-Locke *et al*’s moderate to vigorous physical activity daily guidelines (>7500 steps) from activity while spectating alone, when measured by step count. This is the first study to report PA levels in golf spectators. The self-reported interest in exercise/PA as a reason for attending may be important in explaining the high level of PA achieved. For some, attending the event may represent a deliberate attempt to gain HEPA, while others gain incidental HEPA through their desire to observe particular golfers or the course.^16 17^ Female step count may be lower than male spectators due to factors that may include footwear choice. Equivalent studies of spectator populations’ PA at other tournaments would likely be influenced (positively or negatively) by factors including but not limited to ambient weather conditions, cultural factors, type of tournament and terrain/walkability of the golf course.

### Recommendations for practice/policy and research

Recent strategies from the Department of Culture, Media and Sport and Sport England among others have highlighted the value of spectating at sporting venues and the potential for inspiration and increasing PA.^22 23^ Increasingly, sports organisations/franchises, governing bodies for sport, stadia operators and others are being encouraged to develop practices and policies that promote improved public health for fans and communities. These include efforts relating to healthy eating, alcohol consumption, tobacco use and sustainability as well as promoting PA.

This study confirms it is feasible to study spectator PA and attitudes towards PA in a golf setting. Response rates were good, and compliance rates among participants were exceedingly high. We showed that a reasonable sample size can be achieved with a team of six trained researchers. This will be important information for future work and potential power calculations for sample size requirements. A well-structured questionnaire and collaboration with the tournament organisers are also highly recommended.

Golf spectating does offer an opportunity for PA in this setting and population. Attendance can thus be encouraged, and spectators can be supported to do so in an active fashion in promotional efforts ahead of and during each professional golf event. Golf tournament event planning, marketing efforts, golf course choice and architecture should reflect this. Fans/spectators can receive public health benefits, while tournament organisers/sponsors may realise revenue and corporate and social responsibility benefits. With two-thirds of participants indicating an interest to be more physically active, it may be an opportunity for intervention in a ‘contemplative’ population. While the participants were largely already meeting the guidelines, it should be noted that this is a minimum level of PA and more is better, and that maintenance of PA is critical in adult and ageing populations.

Research priorities for the future include

- Assessing what methods for providing PA information/intervention (eg, big screen, leaflet, poster, email, direct conversation) are welcomed by spectators.
- Investigating whether the spectating experience could be used as a teachable moment to raise awareness of personal PA behaviour, national guidelines and the benefits of PA and influence behavioural change.
- Further study of spectator PA levels in different contexts, and with a larger and more representative sample, which may allow a better estimation of accrued PA, and potential gender and age differences.
- Using qualitative methods to undertake an in-depth exploration of why exercise/PA is valued or not valued by spectators, and exploring the barriers to and facilitators of active spectating at professional golf tournaments among senior tournament decision makers.
- Studying opportunities for other sports/events to explore spectator PA.

### Strengths and limitations

This study was conducted with a pragmatic design and approach.

Strengths include a novel approach in raising awareness of PA through sport and demonstrating public health benefits of sporting events that have thus far been elusive. It also demonstrated the feasibility of conducting research with spectators at professional sporting events in collaboration with event organisers, governing bodies and athlete ambassadors. Research co-produced in this way may help implementation/scale up and assist impact and future intervention delivery in this manner. It is the first to objectively report PA accrued while spectating, while other findings are consistent with previous work describing spectator attitudes to exercise/PA.^15–17^

A number of limitations are evident. Although approximately 600 spectators were approached, those who agreed to wear a pedometer and take part in the study may be more interested in PA and be more physically active than those who declined leading to a selection bias. Observed results may be susceptible to bias; individuals may have modified their responses and behaviours (for example walked more or less) based on what they believe is socially desirable and awareness of their behaviours being observed (Hawthorne effect). Twenty individuals had conclusive proof of pedometer error (for example from GPS/other pedometer), and their step counts were excluded. A smaller number of individuals expressed an opinion that the pedometer had underestimated their step count, but were included due to lack of objective evidence to support, which may have led to an underestimate of their and the observed population’s step count. Step-count data were collected from entry to exit of venue, but did not capture participant PA during the other parts of their day. These limitations and sample size mandate caution in generalising to golf spectators more generally, particularly in different contexts.

## Conclusions

Encouraging people to be more active more often is a public health imperative. A key element of generating increased PA in relation to a sporting event may be to de-emphasise participation in the sport itself and promote PA more generally. Evidence from this study showed that spectators’ rate ‘exercise/physical activity’ as an important reason for attending the golf tournament and that spectating can provide HEPA.
