# Supplementary material for: An observational study of spectators’ step counts and reasons for attending a professional golf tournament in Scotland
Source: BMJ Open Sport Exerc Med. 2017 Jul 21;3(1):e000244. doi: 10.1136/bmjsem-2017-000244 (PMC5530109; doi:10.1136/bmjsem-2017-000244)

**
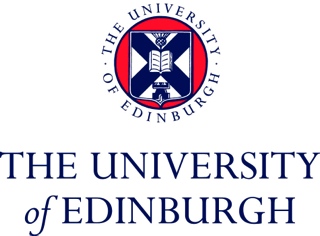
**

**Spectator Questionnaire**

Many thanks for taking the time to fill in this questionnaire. Answers will be used for research only and will not be passed on to any third parties. Your answers will be confidential. We will email you in 4 weeks and ask you to fill a (maximum 5 minute) follow up questionnaire.

| Name |
| --- |
| Email address |
| Contact phone number |
| Date of birth |
| Male / Female |

Number of golf events attended before as a spectator (please circle one)

None 1-2 2-5 5-10 10-20 20-50 over 50

Number of occasions per year when you play golf (please circle one)

None 1-2 2-5 5-10 10-20 20-50 over 50

**Your reasons for spectating.**

**Please rate each reason for importance from 1 (for no importance) to 10 (of extremely high importance).**

1. Watch star players

1 2 3 4 5 6 7 8 9 10

1. Learn from star players

1 2 3 4 5 6 7 8 9 10

1. Non-golfing entertainment

1 2 3 4 5 6 7 8 9 10

1. Atmosphere

1 2 3 4 5 6 7 8 9 10

1. Fresh air

1 2 3 4 5 6 7 8 9 10

1. Exercise/ Physical activity

1 2 3 4 5 6 7 8 9 10

1. Time with friends/ family

1 2 3 4 5 6 7 8 9 10

Please specify below other reasons for spectating (free text)

Thank you for completing this survey


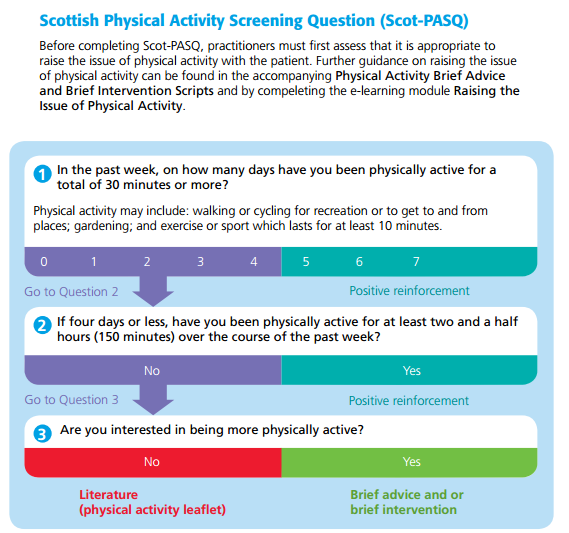

Supplement: Supplementary Appendix 1 [file bmjsem-2017-000244supp001.docx]
